# Maternal Food and Beverage Consumption Behaviors and Discrepant Phthalate Exposure by Race

**DOI:** 10.3390/ijerph18042190

**Published:** 2021-02-23

**Authors:** Mary E. Sterrett, Michael S. Bloom, Erica L. Jamro, Abby G. Wenzel, Rebecca J. Wineland, Elizabeth R. Unal, John Brock, John Kucklick, Kelly Garcia, Roger B. Newman

**Affiliations:** 1 Department of Obstetrics and Gynecology, Medical University of South Carolina, Charleston, SC 29425, USA; msterret@uw.edu (M.E.S.); goodsona@musc.edu (A.G.W.); wineland@musc.edu (R.J.W.); newmanr@musc.edu (R.B.N.); 2 Department of Global and Community Health, George Mason University, Fairfax, VA 22030, USA; kgarcia6@masonlive.gmu.edu; 3 Department of Epidemiology and Biostatistics, University at Albany, State University of New York, Rensselaer, NY 12144, USA; ejamro@albany.edu; 4 Department of Obstetrics and Gynecology, Southern Illinois University School of Medicine, Springfield, IL 62702, USA; eunal@siumed.edu; 5 Department of Chemistry, University of North Carolina Asheville, Asheville, NC 28804, USA; jbrock1@unca.edu; 6 Hollings Marine Laboratory, National Institute of Standards and Technology, Charleston, SC 29412, USA; john.kucklick@nist.gov

**Keywords:** female, phthalic acids/urine, pregnancy, questionnaires, racial groups

## Abstract

*Background*: Differential exposure to endocrine-disrupting chemicals, including phthalate diesters, may contribute to persistent racial/ethnic disparities in women’s reproductive health outcomes. We sought to characterize sources of gestational exposure to these agents that may differ according to maternal race. *Methods*: We enrolled pregnant Black (n = 198), including African American, and White (n = 197) women during the second trimester, and measured eight phthalate monoester metabolites in urine. We assessed confounder-adjusted associations between multiple food and beverage consumption habits, summarized using a principal component analysis, as predictors of maternal urinary phthalate metabolite levels, stratified by race. *Results*: Whites reported significantly greater unprocessed food consumption (42.5% vs. 32.0%; *p* < 0.001) and storage of food in clear unbreakable plastic containers (66.5% vs. 49.3%; *p* < 0.001) than Blacks, while Blacks consumed more canned fruits and vegetables (23.5% vs. 12.2%; *p* < 0.001) than Whites. Using plastics for food storage, microwaving in plastic containers, and using hard plastic water bottles was associated with urinary phthalate concentrations, especially DEHP metabolites (e.g., mean difference = 5.13%; 95% CI: 3.05, 7.25). These associations were driven primarily by Black pregnant women. *Conclusions*: Targeted interventions to reduce maternal exposure to phthalates need to be designed with specific attention to differences in food and beverage consumption behaviors among Black and White women.

## 1. Introduction

Phthalates are plasticizers and solubilizers found in numerous consumer products. They are nearly universally detected in human urine due to widespread exposure from food and beverage contamination as well as inhalation and skin absorption from personal care products [1]. However, there are limited data to characterize potential differences in the sources of phthalate exposure among racially distinct pregnant women. Phthalates cross the placenta and have been found in human amniotic fluid [2]. Multiple phthalates are anti-androgenic endocrine disruptors, potentially causing adverse fetal development during critical time points in gestation [3,4].

Dietary consumption of several foods have been associated with elevated urinary phthalates, notably poultry, seafood, milk, dairy, meat, and egg products [5,6]. Consumption of pre-prepared “fast” foods also appears to be associated with greater phthalate exposure [7]. As mentioned, personal care products are also a critical source of phthalates exposure [8,9,10]. Various occupations have been reported to engender an elevated risk of exposure, including cosmetologists, textile workers, cleaners, workers in the plastics industry, and painters, amongst others [11]. 

Several investigations [12,13], have reported associations between greater gestational phthalates exposure and fetal developmental outcomes, such as anogenital distance [14], preterm birth [15], low birth weight [16], and childhood behavior and executive functioning [17]. Further, we have recently reported heterogeneous associations with fetal developmental endpoints according to maternal race [18,19,20]. These racially discrepant developmental outcomes are found in conjunction with significant differences in phthalate exposure by maternal race between Black and White pregnant women [21]. 

It has been proposed that phthalates and other environmental endocrine disrupting chemicals may be contributing to the significant and persistent racial/ethnic differences in women’s reproductive health outcomes [22]. Therefore, it is important to characterize sources of gestational exposure to these agents that may differ according to maternal race [23]. Furthermore, few data are available to identify phthalate exposure sources among pregnant women from the southeastern United States, who may have different patterns of exposure than pregnant women in other parts of the country [24].

To help to address these existing data gaps, we estimated associations between food and beverage consumption habits and urinary phthalate metabolites in 395 pregnant Black (including African American) and White women from Charleston (South Carolina, USA). These data may allow racial/ethnic specific targeted interventions to limit gestational phthalate exposures and mitigate potential adverse fetal developmental effects. 

## 2. Materials and Methods

### 2.1. Study Population

We enrolled 395 women with singleton pregnancies presenting for routine prenatal ultrasound between 18–22 weeks’ gestation at an urban tertiary level care center. We previously described participant recruitment and enrollment in detail [21]. Women from the Charleston, SC (USA) metropolitan area who planned to deliver at the Medical University of South Carolina (MUSC) between 2011 and 2014 were recruited to participate in this study. Women at least 18 years of age, with an uncomplicated singleton pregnancy dated by a first trimester ultrasound were eligible. Exclusion criteria consisted of pregnancies with an aneuploid or anomalous fetus, use of progesterone or alternative steroids, pregestational diabetes, hyper- or hypothyroidism, or any other known endocrine disorders. All participants completed informed consent and the study protocol was approved by the MUSC Institutional Review Board.

### 2.2. Maternal Urinary Phthalates Analysis

Spot urine specimens were collected in sterile glass jars and transferred to the Hollings Marine Laboratory (Charleston, SC, USA) for analysis. The details of the phthalate analysis and quality control procedures have been previously published [21]. Briefly, following solid phase extraction, urinary phthalate metabolites were determined using an Agilent 1100 Series liquid chromatograph (Agilent 1100, Agilent Technologies, Inc., Santa Clara, CA, USA) coupled to an API 4000 triple quadrupole tandem mass spectrometer (API 4000; Applied Biosystems MDS/Sciex, Foster City, CA, USA). We extracted and analyzed a standard reference material (SRM 3673 Organic Contaminants in Non-Smokers’ Urine) and reagent blank samples in each batch of ten participant samples. If the results for the reference material varied more than three standard deviations from the control, the entire batch was reanalyzed. 

We measured eight prevalent phthalates monoester metabolites reported to have endocrine disrupting activities in 380 women [3], including mono-methyl phthalate (MMP), mono-ethyl phthalate (MEP), mono-n-butyl phthalate (MBP), mono-isobutyl phthalate (MiBP), monobenzyl phthalate (MBzP), mono(2-ethylhexyl) phthalate (MEHP), mono(2-ethyl-5-oxohexyl) phthalate (MEOHP), and mono(2-ethyl-5-hydroxyhexyl) phthalate (MEHHP). As the environment is contaminated with parent phthalate diesters, we measured monoester metabolic products to avoid ubiquitous phthalate quantification [25]. Extraction blank values of 1 mL millipore water extracted were analyzed along with the samples, and those mean blank values (ng/mL) were MMP-0.4095, MEP-0.12, MBP 0.05, MiBP-0.057, MBzP-0.214, MEHP-0.078, MEOHP-0.019, and MEHHP-0.086. We evaluated total diethylhexyl phthalate metabolites (∑DEHP) as the molar sum of MEHP, MEOHP, and MEHHP, and total dibutyl phthalate metabolites (∑DBP) as the molar sum of MBP and MiBP. A composite index of relative anti-androgenic potency factors (∑RPF) was calculated based on toxicological data describing the endocrine disrupting activity of a mixture of phthalate metabolites recognized as anti-androgenic by the National Academies of Science [26]. We defined (∑RPF) as the sum of MBP*1.00, MiBP*0.24, MBzP*0.26, MEHP*0.61, MEHHP*0.61, MEOHP*0.61, and MEP*0.024 as described by Varshavsky and colleagues [27]. We adjusted the spot phthalate concentrations for urine volume using specific gravity (SG) measured using a handheld digital refractometer (Atago USA, Inc., Bellevue, WA, USA), as: Pc = P((1.016 − 1)/(SG − 1)), where Pc was the SG-adjusted phthalate concentration (ng/mL), 1.016 was the mean urinary SG for all participants, and SG was the individual specific gravity of urine [28]. We did not impute data below the method detection limits but rather used the “machine read” values, including negative values, to minimize bias [29,30]. 

### 2.3. Study Questionnaire and Covariate Data

We collected detailed information related to food and beverage consumption habits likely to impact phthalates exposure, including canned foods [31,32], frequency of organic, home grown or unprocessed foods in the diet, frequency and type of plastic containers used for storage or microwaving of food, and use of soft, crushable beverage containers. The survey instrument was used previously in prospective epidemiologic studies of gestational exposure to endocrine disrupting chemicals, including phthalates, and reproductive endpoints [33,34]. Participants were asked about their “typical use in a full day (24 h period)”, referencing the previous week’s use to base their answers on for each dietary question. If not in this format, the structure utilized the following specific responses: “daily”; “not daily, but more than once per week”; “once per week”; “less than once per week”; “rarely”; and “never”. The questionnaire was administered face to face by research staff to participant women upon enrollment. The questions pertained to food and beverage consumption habits associated with exposure to synthetic organic agents, including phthalate diesters, “in general”, to include habits pre-pregnancy and “during pregnancy” specifically. A total of n = 357 women completed the study questionnaire.

Demographic, lifestyle, and occupational information was also obtained by questionnaire, including age (years, continuous), self-identified race (Black or African American, White), body mass index (BMI) calculated from physician-recorded height and weight at time of enrollment (kg/m^2^), household income (<$25,000, $25,000 to $65,000, > $65,000), marital status (married or living as married, and single, including separated, divorced, and widowed), education level (<high school degree, high school graduate or equivalent, some college or technical school, and college graduate or above), and employment status. We categorized employment status according to “high risk“ for phthalate exposure due to frequent use of polyvinyl chloride (PVC) gloves (i.e., cashier/retail worker/sales clerk, food server/processor, hairdresser/cosmetologist, and health care/dental/veterinary workers), and others as “low risk“ for exposure [11,35,36].

### 2.4. Statistical Analysis

We characterized the distributions and frequencies of demographic, lifestyle and occupation factors, urinary phthalate metabolite concentrations, and frequencies of food and beverage consumption habits. We used a natural log transformation to normalize the distribution of urinary phthalates after adding a constant (+3.0) to accommodate negative values. We compared distributions between Black and White pregnant women using Χ^2^-tests and Kruskal Wallis tests as appropriate. We estimated Spearman correlations between food and beverage consumption habits and assessed differences between food and beverage consumption habits “in general” and “during pregnancy” using Friedman tests.

We used multiple linear regression models to simultaneously characterize associations between multiple individual food and beverage consumption habits and maternal urinary phthalate metabolite concentrations, adjusted for age, BMI, marital status, education, and race selected a priori as confounding variables [21]. We adjusted for education as a surrogate of socioeconomic status [37], as there were a large number of missing values for average household income, and education and income were strongly correlated (*p* = 0.62, *p* < 0.0001). We then tested for interaction by incorporating a cross product term between race and each phthalate metabolite and all included covariates in a second set of regression models, which were then stratified by race. This “augmented interaction” approach allows for race-dependent confounding, with a significance test equivalent to comparing stratified effect estimates [38]. We excluded n = 24 (6.3%) with missing questionnaire values.

As questionnaire responses tended to be highly correlated we used principal component analysis (PCA), to summarize the large number of intercorrelated potential predictors (Table S1). This dimension reduction approach [39] grouped congruent food and beverage consumption habit variables into three independent factors, or principal components (PCs), for use as predictors of urinary phthalate metabolites [40]. We employed a polychoric correlation matrix to accommodate the ordinal nature of the questionnaire responses. We selected three PCs based on a scree plot, eigenvalues >1.5, and cumulative explained variance of 53.2%, retaining parsimony. We then simultaneously entered the 3 PCs as predictors into multiple linear regression models using generalized estimating equations to predict individual maternal urinary phthalate metabolite concentrations, adjusted for confounding variables. Finally, we stratified the PCA and regression analyses by race to estimate differences between Black and White pregnant women.

We expressed effect estimates and 95% confidence intervals (95% CI) as the percent difference in maternal urinary phthalate metabolite concentration per incremental exposure unit using (e^β ± 95%CI^-1) × 100. SAS 9.4 (SAS Institute, Inc. Cary, NC, USA) was used for the analysis. Statistical significance was defined as *p* < 0.05 for main effects and *p* < 0.10 for interactions, using 2-tailed tests.

## 3. Results

### 3.1. Study Population

We enrolled 198 (50.1%) Black (including African American) and 197 (49.9%) White pregnant women in the study as depicted in Table 1. Most women used prenatal vitamins (87.8%), and some used prescription or over the counter medications (39.4%). Blacks were 3.1 years younger and had a BMI 3.3 kg/m^2^ higher than Whites on average (*p* < 0.0001). Higher percentages of White women were married (82.5 vs. 24.7%; *p* < 0.0001), had a college education (62.4 vs. 21.1%; *p* < 0.0001), and earned over $65,000 per year (54.4 vs. 4.4%; *p* < 0.0001), compared to Blacks. There was no statistically significant racial difference in participation in designated “high risk” phthalate exposure occupations, including work as a food server/processor, a hair dresser or cosmetologist, or healthcare, including medical, dental, and veterinary workers. Most resided in an urban area but type of housing differed (*p* < 0.0001), in that Whites were more likely to live in a detached single family home than Blacks (63.4% vs. 28.7%) and less likely to live in an apartment (13.4% vs. 44.3%). White and Black women enrolled in the study in similar proportions (*p* = 0.78) in spring (23.5%) summer (32.7%), and fall (25.8%), with fewer in winter (18.0%).

### 3.2. Maternal Urinary Phthalate Concentrations

Table 2 shows the distributions of urinary phthalate metabolites, overall and by maternal race. Phthalates were measured above the limit of detection in at least 93% of urine samples. Blacks had significantly greater concentrations of most urinary phthalate metabolites compared to Whites, excepting MEOHP, MEHHP, and ∑DEHP.

### 3.3. Food and Beverage Consumption Habits

**Figure 1:**
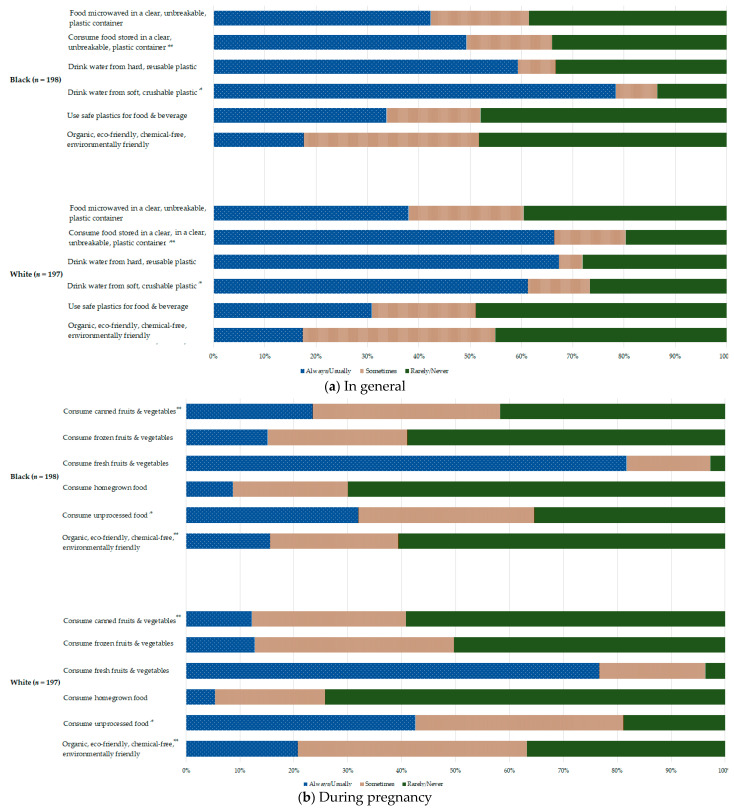
Distribution of food and beverage consumption habits overall and by race, in general (**a**) and during pregnancy (**b**). * *p* < 0.01; ** *p* < 0.001 for difference between races.

Figure 1 shows the distributions of food and beverage consumption habits “in general” (Figure 1a) and “during pregnancy” (Figure 1b), by race. The study questionnaire inquired about habits non-specifically, “in general” (e.g., “I try to make sure it is organic, ecofriendly, chemical-free or environmentally friendly”), and specifically, “during pregnancy” (e.g., “Since you became pregnant, how often have you consumed foods marked ‘organic’, ‘pesticide-free’, or ‘chemical-free’?”). The consumption of organic foods increased for Whites “during pregnancy” relative to “in general” (*p* = 0.04), while consumption of organic foods decreased among Blacks (*p* = 0.002). While income was positively correlated to organic food consumption during pregnancy (r = 0.23, *p* = 0.003), organic food consumption overall was uncorrelated (r = −0.003, *p* = 0.96). Similarly, Whites had a significantly higher frequency of unprocessed food consumption (*p* = 0.002). ”In general”, Blacks were more likely to drink water from a soft crushable plastic container than Whites (*p* = 0.0003), while Whites were more likely to consume food stored in a clear, unbreakable plastic container than Blacks (*p* = 0.001). “During pregnancy”, Black women were more likely to consume canned fruits and vegetables than Whites (*p* = 0.0002). Consumption of organic food and use of safe plastics “in general” were negatively correlated with consumption of canned fruits and vegetables during pregnancy (Table S1).

### 3.4. Urinary Phthalates Associated with Food and Beverage Consumption Behaviors

We simultaneously assessed confounder-adjusted associations between multiple individual food and beverage consumption habits as predictors of maternal urinary phthalate metabolite levels in single models, overall and stratified by race (Table S2). Mutually adjusted, only lower urinary MEP was associated with greater consumption of fresh fruits and vegetables during pregnancy (mean difference = −28.11%; 95% CI: −47.80, −1.00). However, interactions suggested that a greater general use of hard plastic water bottles and consumption of more homegrown and unprocessed foods during pregnancy were associated with higher relative urinary MEP, MMP, ∑DBP, and ∑RPF concentrations among Black women.

We also used PCA to summarize multiple correlated food and beverage consumption habits as three independent PCs. The factor loadings, correlations between each PC and the individual contributing food and beverage consumption habits, varied by race, suggesting heterogeneity in food and beverage consumption habits (Table S3).

As shown in Table S3, when assessing the total study population, Principal Component 1 (PC1) habits included the greatest use of “safe plastics” in general, high organic and unprocessed food intake “in general” and “during pregnancy”, along with frequent consumption of fresh fruits and vegetables during pregnancy. PC1 explained 25.7% of the total variance in food and beverage consumption habits. Principal Component 2 (PC2) habits included frequent use of plastics for food storage, microwaving of food in plastic containers, use of hard plastic water bottles, and infrequent organic food consumption “in general”, explaining 13.6% of the total variance. Principal Component 3 (PC3) habits included consumption of high quantities of canned and frozen fruits and vegetables “during pregnancy”, and the infrequent use of plastic food storage or microwaving of food in plastic containers “in general”, explaining 12.4% of the total variance. Overall, 51.7% of the total variance in food and beverage consumption habits was reflected in these 3 PCs.

As shown in Table 3, more PC1 behavior in the overall study group was associated with greater urinary MBP, MiBP, MEHP, MEOHP, MEHHP, MEP, MMP, ∑DEHP, ∑DBP, and ∑RPF, adjusted for confounders, although with modest effects sizes compared to PC2 habits. PC2 was associated with significantly greater measurements in urinary MEHP, MEOHP, MEHHP, ∑DEHP, and ∑RPF, adjusted for confounders, but with more than twice the magnitude of effect seen in association with PC1. In contrast, the PC3 consumption pattern was associated with lower levels of MBzP and MEP.

The confounder-adjusted associations between the food and beverage consumption habits represented in PC1 and PC2 and urinary phthalate metabolite levels also varied by race. As shown in Table 4, for Whites, greater PC1 was associated with lower levels of MiBP, MMP, and ∑DBP, and greater PC3 was associated with lower levels of MEHP and ∑DEHP. Conversely, a greater PC2 consumption pattern was associated with higher levels of MBP and ∑DBP. As shown in Table 5, both PC1 and PC2 consumption patterns were associated with greater urinary phthalate metabolites among Blacks, whereas a PC3 consumption pattern was not predictive of any urinary phthalate metabolites. PC1 was associated with higher levels of MiBP, MEHP, MEOHP, MEP, and MMP, while greater PC2 was associated with greater MEOHP, MEHHP, and ∑DEHP, and with larger effects sizes than for PC1.

## 4. Discussion

Our findings suggest that three patterns of food and beverage consumption, represented by three distinct PCs, were predictive of urinary phthalate metabolites in 2nd trimester pregnant women, and that the contributing factors differed for White and Black women. We detected urinary phthalate metabolites among 93% of pregnant women, which is consistent with findings from similar exposure assessments that have detected phthalates in 98–100% of pregnant women in both the mainland United States [41,42] and elsewhere [43,44,45,46]. The race stratified PCA results suggested differences in the important contributing exposure sources to the PCs among White and Black pregnant women. As Rudel et al. [47] showed dietary replacement can reduce exposure concentrations, our results further suggest that different dietary sources or behaviors may be more relevant sources of phthalate exposure for some groups. To our knowledge, this is the first study to report differences in gestational phthalate exposure by food and beverage consumption habits in Black and White women conceiving spontaneously.

Similar dietary consumption patterns were associated with different urinary phthalate concentrations among pregnant Black and White women. Food and beverage consumption habits are known to correlate with socioeconomic factors and race [48]. This may reflect in part the differential loadings of individual food and beverage consumption questions on PCs between Black and White pregnant women. For example, PC1 in Whites mostly reflected consumption of organic foods, use of “safe plastics”, and consumption of unprocessed food, fresh fruits and vegetables during pregnancy and was associated with lower phthalate metabolite concentrations. However, in Blacks, PC1 also reflected use of hard plastic water bottles and consumption of frozen fruits and vegetables during pregnancy, which was associated with higher phthalate metabolite concentrations. It is also possible that our survey instrument may have failed to thoroughly distinguish between consumption patterns that differ between the races or were not detected. Given these concerns, dietary habits deserve further investigation in a future study utilizing a more racially specific questionnaire tool.

There was a statistically significant difference in the average sum of MBP and MiBP (nmol/mL ΣDBP) for Black pregnant women at 183.5 nmol/L compared to 97.9 nmol/L for White pregnant women. This ΣDBP represents exposure to phthalates commonly used in personal care products [49]. In contrast, when we assessed ΣDEHP exposure, more commonly representative of plasticizers in polyvinyl chloride plastics, no significant difference was found [48]. Non-occupational exposure to high molecular weight phthalates, like DEHP, occurs most commonly in food consumption [50]. Other results suggest that high consumption of organic and unprocessed food [51] and use of safe plastics [47] were associated with lower plastics-related phthalate exposure. A meta-analysis of 10 studies of dietary predictors of phthalates exposure during pregnancy reported that use of plastic containers was associated with higher urinary phthalate metabolites [52], but did not delineate which metabolites were elevated amongst all the studies when the data was grouped together. Healthier food choices, such as consumption of organic or home grown/raised/caught foods, were associated with lower urinary phthalate levels in that meta-analysis [52].

PC1 captured a pattern of food and beverage consumption habits, which might be contemporaneously viewed as “healthy”, including a preference for organic foods, safe plastics, and fresh foods and vegetables. Previous studies have evaluated socially separate groups, such as Old Order Mennonites, and found significantly lower urinary phthalate metabolite levels compared to a nationally representative sample of pregnant U.S. women [32]. These lower exposures were attributed to consumption of home-grown food, consuming few processed foods, and use of fewer household chemicals and personal care products. A dietary intervention study also demonstrated reduced concentrations of urinary DEHP metabolites following the introduction of a fresh foods diet that were not canned or packaged [47]. Sathyanarayana et al. (2013) found an unexpected increase of DEHP metabolites in a similar intervention study, which involved a complete dietary replacement with fresh and organic foods prepared without plastics [53]. The authors hypothesized that specific spices and dairy products in the dietary replacement intervention group had high levels of DEHP, potentially causing the unexpected phthalate spike, despite controlling for plastic packaging exposure. Their results found that decreasing plastics exposure did not necessarily decrease high molecular weight phthalate exposure [53]. These discrepant findings across studies indicate a need for further investigation.

A PC1 consumption pattern in Whites was associated with lower levels of MiBP and MMP. However, similar PC1 behavior was more strongly associated with urinary MiBP, MEHP, MEOHP, MEP and MMP among Black women. This might be explained in part by the different factor loadings, in which PC1 reflected greater use of plastic water and food containers (both soft and hard plastics as well as microwaving in plastic) among Blacks than Whites. In light of studies finding that controlling for plastic exposure and packaging does not always lead to a decrease in high molecular weight phthalate, examining broader patterns and habits of consumption is important [53]. A more comprehensive dietary assessment will be necessary for a more definitive interpretation of this result.

PC2 behaviors, characterized by food storage and microwaving in plastics, use of hard plastic water bottles, and little organic food consumption “in general”, correlated with a greater concentration of urinary ∑DEHP as expected [50,54], and the association with ∑RPF was twice as strong as for PC1 dietary behaviors. Research on specific food exposures within racial groups found diet to be the major source of DEHP [4,6,32,50,55]. However, we found that the PC2-∑DEHP association appeared to be primarily in Black women, for whom PC2 consumption patterns were correlated to greater MEOHP and MEHHP, whereas there was no association with DEHP metabolites among White women. The factor loadings suggested a greater use of plastic food storage and microwaving in plastic in the PC2 food and beverage consumption habits. Urinary ∑RPF, a composite relative anti-androgenic potency phthalate exposure variable, was significantly higher in Black pregnant women compared to White pregnant women in our study, as has been previously reported [27]. Our results are consistent with others showing differences in exposure according to race/ethnicity, specifically regarding DEHP [48,56].

PC3 most closely reflected consumption of higher quantities of canned and frozen fruits and vegetables while pregnant, and infrequent use of plastic food storage in general. Greater PC3 behaviors were associated with less MEHP, and therefore ΣDEHP, in White pregnant women. However, PC3 behaviors in Black pregnant women showed no significant difference in MEHP, ΣDEHP, or any other phthalate exposures. Again, PC3 factor loadings indicated different consumption habits among White pregnant women, use of “safe” plastics in general and organic foods during pregnancy, not indicated for Black pregnant women.

Our data support differing phthalate exposure by race, yet the limited correlation of our captured food and beverage consumption habits implies alternative sources of phthalate exposure. However, when assessing differences between self-identified race cohorts, researchers and policy makers must be cognizant that ”race” itself implies a sociocultural group, and not a biologic difference [57]. Structural forces including access to health care, food sources and security, housing, employment, and other factors that condition living very likely have a significant impact on phthalate exposure, and could not be captured in this study [58,59].

Our diverse study population enabled us to identify different phthalate exposure behaviors in Black and White pregnant women, and our mixtures-based approach using PCA integrated multiple correlated food and beverage consumption habits to reflect human behavior more in profile than can be achieved using a more traditional reductionist strategy. We also used a urine biomarker to objectively assess phthalate exposure in pregnant women and adjusted for a comprehensive panel of covariates to mitigate confounding. However, this was a spot urine sample, and phthalate metabolites have a short half-life [60], which may have misclassified outcomes for some women. Furthermore, we did not conduct a 24-h dietary recall, which may also have misclassified exposure.

Assessing phthalate exposure with the inclusion of additional behaviors such as personal care products could identify other sources of phthalate exposure amenable to reduction during pregnancy. Unfortunately, we did not collect specifics of personal care product use information, leaving us unable to directly address the hypothesis that personal care products contributed to observed disparities [22,23,61], and may have misclassified exposure for some women. For instance, the active chemicals in hair products primarily used by Black women may be a major source of exposure to phthalate diesters resulting in misclassified exposure among our Black participants [23,62]. We were unable to include medication use, which may be a source of exposure, potentially misclassifying exposure for some women [63]. We also did not incorporate season of enrollment, a predictor of urinary phthalates [64], although similar between Black and White pregnant women in our study. Women may also have inadequate recall or interpretation of “safe” plastics and manufacturer practices may vary [65].

Although we adjusted for maternal education, it would also be beneficial to match Black and White women by household income and employment type, as socioeconomic status has known impact on DEHP metabolites [48]. Food availability sources such as governmental food assistance is also likely to be different among racial groups. Our study questionnaire was also lacking in its exploration of specific food types consumed. Investigators report varying DEHP levels with different foods, specifically poultry, cooking oils, and cream-based dairy products [5] or for differing intake of high fat foods [7].

Despite our moderate sample size, we may have been insufficiently powered to detect modest associations in the overall study population. Our sample size furthermore did not allow us to test race-based interactions in the confounder-adjusted PCA models, given three factors. We were also unable to assess the impact of race/ethnicity other than Black and White in our study. A larger, adequately powered investigation with a more comprehensive capture of dietary factors in the past 24 h and lifestyle factors and a more diverse profile of study participants will be necessary to achieve more definitive results.

## 5. Conclusions

Our study results suggest there are racial differences in the sources of maternal phthalate exposure. In Whites, adoption or avoidance of specific dietary factors had anticipated associations with greater or lesser gestational urinary phthalate metabolites. However, in Black, including African American, women it did not appear that anticipated ”healthier” behavior patterns were associated with lower exposure. The results indicate that presumably protective dietary habits such as the use of safe plastics, high organic and unprocessed food intake, and frequent consumption of fresh fruits and vegetables while pregnant cannot necessarily be applied regardless of race and be expected to have equivalent effects on phthalate exposures. We cannot rule out the possibility of differences in phthalate metabolism, or perhaps physiological differences [66]. This is not to say that phthalate exposure in dietary habits is driven by different physiology, but that we suspect the broader social and dietary habits within a racial group contribute to phthalate exposure. Future work is needed to test these findings while incorporating other potential exposure behaviors such as personal care product use assessed through the lens of critical race theory. These study results will be helpful in the continuing effort to explain the racial disparities seen in reproductive outcomes and to limit gestational exposure to phthalate chemicals.

## Figures and Tables

**Table 1:** Distributions of demographic and occupational characteristics overall and by race.

Characteristic	Overall (n = 395)		White (n = 197)		Black (n = 198)		*p* Value
Age, mean (SD) ^a^	27.4	5.6	29.0	5.2	25.9	5.6	<0.0001
BMI, mean (SD) ^b^	29.3	7.4	27.6	6.3	30.9	8.1	<0.0001
Education, n (%) ^c^							<0.0001
< High school	41	11.0%	12	6.4%	29	15.7%	-
High school	80	21.4%	19	23.8%	61	33.0%	-
Some college	96	25.7%	40	21.2%	56	30.3%	-
Finish college	101	27.0%	73	38.6%	28	15.1%	
Graduate work	56	15.0%	45	23.8%	11	6.0%	-
Annual household income, n (%) ^d^							<0.0001
<$25k	93	34.3%	20	12.7%	73	64.6%	-
$25k–$65k	87	32.1%	52	32.9%	35	31.0%	-
>$65k	91	33.4%	86	54.4%	5	4.4%	-
Marital status, n (%) ^e,f^							<0.0001
Married	202	53.9%	156	82.5%	46	24.7%	-
Single	173	46.1%	33	17.5%	140	75.3%	-
Job, n (%) ^g,h^							0.36
High risk	101	34.8%	58	37.2%	43	32.1%	-
Low risk	189	65.2%	98	62.8%	91	67.9%	-
Housing, n(%) ^i^							
Detached single family home	172	46.4%	119	63.4%	53	28.7%	<0.0001
Attached single family home	36	9.7%	17	9.1%	19	10.3%	-
Apartment	107	28.8%	25	13.4%	82	44.3%	-
Mobile home/trailer	36	9.7%	17	9.1%	19	10.3%	-
Condminium	7	1.9%	5	2.7%	2	1.1%	-
Other	13	3.5%	3	1.6%	10	5.4%	-
Season enrolled, n(%) ^j^							
Winter	36	18.0%	36	18.3%	35	17.7%	0.78
Spring	44	23.5%	44	22.3%	49	24.8%	-
Summer	62	32.7%	62	31.5%	67	33.8%	-
Fall	55	25.8%	55	27.9%	47	23.7%	-

^a^ n = 1 missing value; ^b^ n = 3 missing values; ^c^ n = 21 missing values; ^d^ n = 124 missing values; ^e^ Married includes married and living as married, Single includes single, separated, divorced, widowed; ^f^ n = 20 missing values; ^g^ High exposure jobs (food server/processor, hairdresser/cosmetologist, healthcare/dental/veterinary workers) vs. all other jobs; ^h^ n = 105 missing values; ^i^ n = 24 missing values; ^j^ Winter includes December, January, and February, spring includes March, April, and May, summer includes June, July, and August, and fall includes September, October, and November.

**Table 2:** Geometric mean (95% confidence interval) concentrations of specific-gravity adjusted phthalates during the 2nd trimester (ng/mL).

Phthalate Metabolite	Overall (n = 380)	White (n = 193)	Black (n = 187)	*p* Value ^a^
	GM (95% CI)	GM (95% CI)	GM (95% CI)	
MBP	17.4 (16.0, 19.0)	13.2 (11.7, 14.8)	23.0 (20.4, 25.8)	<0.0001
MiBP	12.4 (11.3, 13.5)	8.9 (7.9, 9.9)	17.1 (15.2, 19.1)	<0.0001
MBzP	13.4 (11.9, 15.0)	9.2 (7.7, 10.9)	19.2 (16.7, 22.0)	<0.0001
MEHP	3.9 (3.5, 4.3)	3.4 (2.9, 4.0)	4.4 (3.8, 5.0)	0.02
MEOHP	6.6 (6.1, 7.2)	6.5 (5.8, 7.3)	6.7 (6.0, 7.5)	0.72
MEHHP	8.3 (7.6, 9.0)	8.0 (7.1, 9.0)	8.6 (7.6, 9.7)	0.39
MEP	59.6 (51.6, 68.6) ^b^	39.5 (32.5, 47.8)	90.4 (74.3, 109.9) ^b^	<0.0001 ^b^
MMP	2.9 (2.5, 3.3) ^b^	2.1 (1.6, 2.7)	3.8 (3.3, 4.3) ^b^	<0.0001 ^b^
ΣDEHP	61.1 (56.4, 66.2)	58.5 (52.3, 65.4)	63.8 (56.8, 71.6)	0.29
ΣDBP	133.5 (122.8, 145.1)	97.9 (87.7, 109.1)	183.5 (164.4, 204.9)	<0.0001
ΣRPF	41.7 (38.6, 45.0) ^b^	32.8 (29.4, 36.5)	53.3 (48.1, 58.9) ^b^	<0.0001 ^b^

^a^ Mann-Whitney U-test; ^b^ n = 1 missing value; ΣDEHP, sum of MEHP, MEOHP, and MEHHP in nmol/L; ΣDBP, sum of MBP and MiBP in nmol/L; ΣRPF, sum of MBP, MiBP, MBzP, MEHP, MEHHP, MEOHP, and MEP in µg/L weighted by relative anti-androgen potency factors (RPF).

**Table 3:** Percent difference in specific gravity-adjusted urinary phthalate metabolites associated with principal components (PC) representing food and beverage consumption habits, among all pregnant women (n = 356) ^a^.

Phthalate Metabolite	PC Factor 1:		PC Factor 2:		PC Factor 3:	
	% Difference (95% CI)	*p* Value	% Difference (95% CI)	*p* Value	% Difference (95% CI)	*p* Value
MBP	**0.50 (0.20, 1.01)**	**0.01**	1.01 (−1.00, 3.05)	0.23	−1.98 (−6.76, 3.05)	0.37
MiBP	**1.01 (1.01, 1.01)**	**<0.0001**	1.01 (−0.10, 2.02)	0.07	−2.96 (−6.76, 2.02)	0.22
MBzP	0.20 (−0.20, 1.01)	0.26	1.01 (−1.00, 3.05)	0.36	**−7.69 (−13.06, −1.98)**	**0.01**
MEHP	**1.01 (1.01, 2.02)**	**<0.0001**	**2.02 (1.01, 3.05)**	**<0.0001**	2.02 (−1.98, 6.18)	0.41
MEOHP	**1.02 (0.30, 1.01)**	**<0.0001**	**2.02 (1.01, 3.05)**	**<0.0001**	0.40 (−3.92, 4.08)	0.84
MEHHP	**0.40 (0.10, 1.01)**	**0.01**	**2.02 (1.01, 3.05)**	**<0.0001**	1.01 (−3.92, 6.18)	0.66
MEP ^b^	**3.05 (3.05, 4.08)**	**<0.0001**	1.01 (−2.96, 5.13)	0.75	**−10.42 (−19.75, −0.30)**	**0.04**
MMP ^b^	**0.40 (0.10, 1.01)**	**0.01**	0.50 (−1.00, 2.02)	0.58	−4.88 (−9.52, 1.01)	0.09
∑DEHP	**2.02 (1.01, 3.05)**	**<0.0001**	**5.13 (3.05, 7.25)**	**<0.0001**	2.02 (−4.88, 9.42)	0.57
∑DBP	**1.01 (0.40, 1.01)**	**<0.0001**	1.01 (−0.40, 2.02)	0.17	−2.96 (−7.69, 2.02)	0.21
ΣRPF ^b^	**1.01 (1.01, 1.01)**	**<0.0001**	**2.02 (0.03, 3.05)**	**0.05**	−1.98 (−5.82, 2.02)	0.35

NOTE: PC 1 corresponds to high organic and unprocessed food intake, the high consumption of fresh fruits and vegetables while pregnant, and the use of safe plastics “in general”; PC 2 corresponds to use of plastics for food storage, microwaving of plastic containers, use of hard plastic water bottles, and little organic food consumption “in general”; PC 3 corresponds to eating canned and frozen fruits and vegetables while pregnant, and the infrequent use of plastic food storage “in general”; *p* < 0.05 in bold typeface. ^a^ Adjusted for age (years), BMI (kg/m^2^), marital status (married or living as married vs. single (single, separated, divorced, widowed)), education (<high school, high school, some college, ≥college), and race (White vs. Black). ^b^ n = 1 missing value. ΣDEHP, sum of MEHP, MEOHP, and MEHHP in nmol/L; ΣDBP, sum of MBP and MiBP in nmol/L; ΣRPF sum of MBP, MiBP, MBzP, MEHP, MEHHP, MEOHP, and MEP in µg/L weighted by relative anti-androgen potency factors (RPF).

**Table 4:** Percent difference in specific gravity-adjusted urinary phthalate metabolites associated with principal components (PC) representing food and beverage consumption habits, among White pregnant women (n = 182) ^a^.

Phthalate Metabolite	PC Factor 1:		PC Factor 2:		PC Factor 3:	
	% Difference (95% CI)	*p* Value	% DiFference (95% CI)	*p* Value	% Difference (95% CI)	*p* Value
MBP	−1.00 (−1.00, 0.00)	0.05	**5.13 (1.01, 8.33)**	**0.01**	2.02 (−1.00, 5.13)	0.22
MiBP	**−1.00 (−1.00, −0.20)**	**0.01**	3.05 (−0.30, 6.18)	0.07	1.01 (−2.96, 5.13)	0.74
MBzP	−0.10 (−2.96, 3.05)	0.93	4.08 (−2.96, 10.52)	0.24	−1.98 (−6.76, 3.05)	0.52
MEHP	1.01 (−1.98, 3.05)	0.59	−0.30 (−2.96, 2.02)	0.83	**−3.92 (−6.76, −1.00)**	**0.01**
MEOHP	1.01 (−1.98, 3.05)	0.68	0.00 (−2.96, 3.05)	1.00	−1.98 (−6.76, 2.02)	0.34
MEHHP	1.01 (−1.98, 3.05)	0.49	−1.00 (−3.92, 2.02)	0.48	−2.96 (−6.76, 2.02)	0.28
MEP	−1.00 (−2.96, 1.01)	0.48	5.13 (−3.92, 15.03)	0.28	−1.00 (−12.19, 11.63)	0.88
MMP	**−1.98 (−3.92, −0.04)**	**0.05**	3.05 (−3.92, 10.52)	0.36	3.05 (−2.96, 8.33)	0.33
∑DEHP	1.01 (−1.98, 5.13)	0.56	−1.00 (−4.88, 3.05)	0.64	**−5.82 (−8.61, −1.98)**	**0.01**
∑DBP	**−1.00 (−1.98, −0.30)**	**0.01**	**5.13 (1.01, 9.42)**	**0.01**	2.02 (−1.98, 7.25)	0.34
ΣRPF	0.10 (−1.00, 2.02)	0.86	3.05 (−0.50, 6.18)	0.10	−1.00 (−4.88, 2.02)	0.48

NOTE: PC 1 corresponds to high organic and unprocessed food intake, consumption of fresh fruits and vegetables while pregnant, and the use of safe plastics “in general”; PC 2 corresponds to use of plastics for food storage, microwaving of plastic containers, use of hard plastic water bottles, and little organic food consumption “in general”; PC 3 corresponds to eating canned and frozen fruits and vegetables while pregnant, and the infrequent use of plastic food storage “in general”; *p* < 0.05 in bold typeface. ^a^ Adjusted for age (years), BMI (kg/m^2^), marital status (married or living as married vs. single (single, separated, divorced, widowed)), and education (< high school, high school, some college, ≥ college). ΣDEHP, sum of MEHP, MEOHP, and MEHHP in nmol/L; ΣDBP, sum of MBP and MiBP in nmol/L; ΣRPF sum of MBP, MiBP, MBzP, MEHP, MEHHP, MEOHP, and MEP in µg/L weighted by relative anti-androgen potency factors (RPF).

**Table 5:** Percent difference in specific gravity-adjusted urinary phthalate metabolites associated with principal components (PC) representing food and beverage consumption habits, among Black pregnant women (n = 174).

Phthalate Metabolite	PC Factor 1:		PC Factor 2:		PC Factor 3:	
	% Difference (95% CI)	*p* Value	% Difference (95% CI)	*p* Value	% Difference (95% CI)	*p* Value
MBP	2.02 (−1.00, 5.13)	0.29	0.30 (−2.96, 4.08)	0.87	2.02 (−7.69, 12.75)	0.70
MiBP	**1.01 (0.30, 2.02)**	**0.01**	0.30 (−1.98, 3.05)	0.81	−2.96 (−11.31, 6.18)	0.51
MBzP	1.01 (−1.00, 2.02)	0.31	−1.00 (−4.88, 3.05)	0.67	5.13 (−4.88, 17.35)	0.32
MEHP	**1.01 (0.10, 3.05)**	**0.03**	2.02 (−0.10, 5.13)	0.05	−1.98 (−10.42, 6.18)	0.60
MEOHP	**1.01 (0.10, 1.01)**	**0.03**	**2.02 (1.01, 4.08)**	**0.01**	−1.00 (−8.61, 7.25)	0.78
MEHHP	0.30 (−0.30, 1.01)	0.32	**3.05 (1.01, 5.13)**	**0.01**	−1.98 (−10.42, 6.18)	0.68
MEP ^b^	**4.08 (3.05, 6.18)**	**<0.0001**	−1.00 (−7.69, 6.18)	0.81	15.03 (−4.88, 39.10)	0.14
MMP ^b^	**0.50 (0.00, 1.01)**	**0.05**	−1.00 (−2.96, 2.02)	0.66	4.08 (−2.96, 12.75)	0.25
∑DEHP	1.01 (−0.10, 2.02)	0.08	**4.08 (1.01, 7.25)**	**0.01**	−1.00 (−12.19, 11.63)	0.86
∑DBP	2.02 (−1.00, 4.08)	0.17	0.30 (−2.96, 4.08)	0.89	0.30 (−10.42, 11.63)	0.95
ΣRPF ^b^	2.02 (−0.20, 4.08)	0.07	1.01 (−3.92, 6.18	0.64	2.02 (−7.69, 11.63)	0.73

NOTE: PC 1 corresponds to high organic and unprocessed food intake, consumption of fresh fruits and vegetables while pregnant, and the use of safe plastics “in general”; PC 2 corresponds to use of plastics for food storage, microwaving of plastic containers, use of hard plastic water bottles, and little organic food consumption “in general”; PC 3 corresponds to eating canned and frozen fruits and vegetables while pregnant, and the infrequent use of plastic food storage “in general”; *p* < 0.05 in bold typeface. ^a^ Adjusted for age (years), BMI (kg/m^2^), marital status (married or living as married vs. single (single, separated, divorced, widowed)), and education (< high school, high school, some college, ≥ college); ^b^ n = 1 missing value. ΣDEHP, sum of MEHP, MEOHP, MEHHP in nmol/L; ΣDBP, sum of MBP and MiBP in nmol/L; ΣRPF weighted sum of MBP, MiBP, MBZP, MEHP, MEHHP, MEOHP, and MEP in µg/L weighted by relative anti-androgen potency factors (RPF).

## Data Availability

The data presented in this study are available on request from the corresponding author. The data are not publicly available due to privacy.

## Supplementary Materials

The following are available online at https://www.mdpi.com/1660-4601/18/4/2190/s1, Table S1: Correlation coefficients for food and beverage consumption in general and during pregnancy, Table S2: Percent difference (95% confidence interval) in urinary phthalates associated with individual food and beverage consumption habits, by race, Table S3. Principal component analysis factor loadings.
