# Supplementary material for: Maternal Food and Beverage Consumption Behaviors and Discrepant Phthalate Exposure by Race
Source: Int J Environ Res Public Health. 2021 Feb 23;18(4):2190. doi: 10.3390/ijerph18042190 (PMC7927108; doi:10.3390/ijerph18042190)
Supplement: Supplementary file 1 [file ijerph-18-02190-s001.pdf]

Table S1. Correlation coefficients for food and beverage consumption in general and during pregnancy.

		Food & beverage consumption in general						Food & beverage consumption during pregnancy					
		Organic	Safe Plastics	Water: Soft Plastic	Water: Hard Plastic	Plastic Food Storage	Microwave Plastic	Organic	Unprocessed food	Home grown food	Fresh Fruits & Veg	Frozen Fruits & Veg	Canned Fruits & Veg
**In general**	Organic	-	**0.44**	**−0. 13**	0.08	−0.11	**−0.15**	**0.60**	**0.27**	**0.20**	**0.17**	0.05	**−0.14**
	Safe Plastics	<0.0001	-	**−0.11**	**0.20**	−0.05	−0.07	**0.37**	**0.17**	**0.20**	**0.21**	0.03	**−0.13**
	Water: Soft Plastic	0.02	0.05	-	−0.01	0.05	0.06	−0.11	**−0.12**	0.06	0.002	−0.05	**0.11**
	Water: Hard Plastic	0.19	0.001	0.83	-	**0.22**	0.04	**0.12**	0.07	0.01	0.09	**0.12**	0.03
	Plastic Food Storage	0.06	0.36	0.35	0.001	-	**0.25**	0.03	0.05	−0.004	0.01	0.06	**−0.15**
	Microwave Plastic	0.01	0.17	0.23	0.46	<0.0001	-	−0.05	0.03	0.01	−0.06	0.09	0.07
**During pregnancy**	Organic	<0.0001	<0.0001	0.06	0.05	0.66	0.36	-	**0.26**	**0.17**	**0.22**	**0.11**	**−0.22**
	Unprocessed food	<0.0001	0.001	0.03	0.22	0.38	0.57	<0.0001	-	0.07	**0.21**	0.02	**−0.19**
	Home grown food	0.0002	0.0001	0.24	0.82	0.95	0.83	0.003	0.18	-	0.08	**0.11**	0.02
	Fresh Fruits & Veg	0.001	<0.0001	0.97	0.14	0.89	0.22	<0.0001	<0.0001	0.12	-	**−0.11**	**−0.30**
	Frozen Fruits & Veg	0.36	0.55	0.36	0.04	0.26	0.08	0.04	0.67	0.04	0.03	-	**0.12**
	Canned Fruits & Veg	0.01	0.02	0.03	0.64	0.01	0.18	<0.0001	0.00	0.77	<0.0001	0.02	-

NOTE: Spearman correlation coefficients found above the diagonal, *p*-values for correlation found below the diagonal; correlations with $p < 0.05$ in bold typeface.

Table S2. Percent difference (95% confidence interval) in gestational urinary phthalates associated with food and beverage consumption habits, by race.[a]

	MBP			MiBP		
	Overall (n = 395)	White (n = 197)	Black (n = 198)	Overall (n = 395)	White (n = 197)	Black (n = 198)
**Food & beverage consumption in general**						
Organic	−6.76 (−17.30, 4.08)	−12.19 (−26.66, 4.08)	−4.88 (−17.30, 9.42)	−2.96 (−13.93, 9.42)	4.08 (−11.31, 221.4)	1.01 (−13.06, 17.35)
Safe Plastics	6.18 (−4.88, 17.35)	3.05 (−12.19, 20.92)	10.52 (−2.96, 27.12)	2.02 (−8.61, 13.88)	10.52 (−3.92, 27.12)	5.13 (−8.61, 20.92)
Water: Soft Plastic	7.25 (−2.96, 19.72)	2.02 (−11.31, 17.35)	4.08 (−11.31, 20.92)	8.33 (−2.96, 19.72)	−5.82 (−18.13, 8.33)	−1.98 (−17.30, 16.18)
Water: Hard Plastic	−0.99 (−9.52, 9.42)	−7.69 (−19.75, 5.13)	11.63 (−1.00, 27.12)	−0.99 (−10.42, 9.42)	13.88 (−1.98, 33.64)	9.42 (−4.88, 25.86)
Plastic Food Storage	4.08 (−6.76, 15.03)	6.18 (−8.61, 24.61)	0.00 (−12.19, 12.75)	9.42 (−1.98, 20.92)	−4.88 (−17.30, 9.42)	1.01 (−12.19, 16.18)
Microwave Plastic	−4.88 (−13.93, 5.13)	−6.76 (−18.94, 8.33)	−8.61 (−19.75, 4.08)	−4.88 (−13.93, 5.13)	1.01 (−11.31, 16.18)	−5.82 (−18.13, 8.33)
**Food & beverage consumption during pregnancy**						
Organic	−1.98 (−11.31, 9.42)	−0.99 (−13.06, 13.88)	0.00 (−13.93, 16.18)	−1.98 (−11.31, 8.33)	−8.61 (−19.75, 4.08)	−3.92 (−16.47, 10.52)
Unprocessed Food	6.18 (−3.92, 16.18)	−1.98 (−14.79, 11.63)	17.35 (2.02, 33.64)	0.00 (−8.61, 9.42)	−7.69 (−21.34, 9.42)	8.33 (−4.88, 22.14)
Home Grown Food	5.13 (−6.76, 18.53)	−4.88 (−19.75, 12.75)	10.52 (−6.76, 29.69)	8.33 (−2.96, 20.92)	−7.69 (−25.17, 13.88)	19.72 (2.02, 39.10)
Fresh Fruits & Veg	−5.82 (−19.75, 11.63)	−5.82 (−23.66, 17.35)	−4.88 (−25.17, 20.92)	−10.42 (−22.89, 4.08)	−1.98 (−13.93, 11.63)	−11.31 (−28.82, 10.52)
Frozen Fruits & Veg	−9.52 (−18.94, 0.40)	−8.61 (−20.55, 4.08)	−10.42 (−22.89, 5.13)	0.00 (−9.52, 9.42)	1.01 (−13.06, 18.53)	0.00 (−13.06, 15.03)
Canned Fruits & Veg	1.01 (−9.52, 12.75)	12.75 (−2.96, 32.31)	−6.76 (−18.94, 6.18)	−4.88 (−13.93, 5.13)	0.00 (0.00, 0.00)	−7.69 (−18.94, 5.13)

NOTE: Grey shading indicates $p < 0.10$ for interaction by race. MBP, mono-n-butyl phthalate; MiBP, mono-isobutyl phthalate.

[a] Adjusted for age (years), BMI (kg/m^2), marital status (married or living as married vs. single (single, separated, divorced, widowed)), and education (< High school, High school, some college, ≥ college).

Table S2. Percent difference (95% confidence interval) in gestational urinary phthalates associated with food and beverage consumption habits, by race.[a]

	MBzP			MEHP		
	Overall (*n* = 395)	White (*n* = 197)	Black (*n* = 198)	Overall (*n* = 395)	White (*n* = 197)	Black (*n* = 198)
**Food & beverage consumption in general**						
Organic	−5.82 (−20.55, 11.63)	−27.39 (−42.88, −6.76)	18.53 (−2.96, 43.33)	−3.92 (−13.93, 7.25)	−9.52 (−22.12, 5.13)	1.01 (−13.06, 16.18)
Safe Plastics	0.00 (−13.93, 16.18)	12.75 (−10.42, 40.49)	−6.76 (−22.12, 12.75)	4.08 (−5.82, 15.03)	0.00 (−13.06, 15.03)	13.88 (−0.20, 31.00)
Water: Soft Plastic	11.63 (−3.92, 29.69)	10.52 (−9.52, 34.99)	−1.98 (−21.34, 22.14)	4.08 (−5.82, 13.88)	2.02 (−9.52, 15.03)	6.18 (−9.52, 24.61)
Water: Hard Plastic	9.42 (−4.88, 24.61)	−1.98 (−18.94, 18.53)	27.12 (6.18, 52.20)	3.05 (−5.82, 12.75)	3.05 (−8.61, 16.18)	1.01 (−11.31, 15.03)
Plastic Food Storage	−5.82 (−18.94, 9.42)	−3.92 (−22.12, 19.72)	−5.82 (−21.34, 12.75)	−2.96 (−12.19, 6.18)	−1.98 (−13.93, 11.63)	−6.76 (−18.13, 6.18)
Microwave Plastic	7.25 (−7.69, 23.37)	9.42 (−11.31, 33.64)	−0.99 (−17.30, 18.53)	0.00 (−8.61, 10.52)	2.02 (−9.52, 15.03)	−3.92 (−15.63, 10.52)
**Food & beverage consumption during pregnancy**						
Organic	5.13 (−7.69, 20.92)	−2.96 (−21.34, 18.53)	17.35 (−1.00, 40.49)	−2.96 (−11.31, 6.18)	−7.69 (−18.94, 4.08)	4.08 (−9.52, 20.92)
Unprocessed Food	7.25 (−4.88, 22.14)	4.08 (−15.63, 27.12)	17.35 (1.01, 37.71)	3.05 (−4.88, 12.75)	2.02 (−9.52, 16.18)	5.13 (−7.69, 19.72)
Home Grown Food	1.01 (−13.93, 18.53)	2.02 (−20.55, 32.31)	−4.88 (−21.34, 15.03)	4.08 (−6.76, 15.03)	3.05 (−12.19, 20.92)	2.02 (−12.19, 18.53)
Fresh Fruits & Veg	−18.94 (−34.30, 1.01)	−12.19 (−36.24, 20.92)	−29.53 (−46.74, 7.69)	4.08 (−10.42, 19.72)	5.13 (−13.93, 28.40)	−1.00 (−20.55, 23.37)
Frozen Fruits & Veg	−8.61 (−20.55, 5.13)	−10.42 (−26.66, 10.52)	−3.92 (−19.75, 15.03)	−1.98 (−11.31, 7.25)	−5.82 (−17.30, 7.25)	2.02 (−12.19, 17.35)
Canned Fruits & Veg	7.25 (−6.76, 23.37)	20.92 (−4.88, 53.73)	−2.96 (−17.30, 13.88)	−1.98 (−10.42, 8.33)	4.08 (−10.42, 20.92)	−5.82 (−17.30, 7.25)

NOTE: Grey shading indicates $p < 0.10$ for interaction by race. MBzP, monobenzyl phthalate; MEHP, mono(2-ethylhexyl) phthalate.

[a] Adjusted for age (years), BMI (kg/m^2), marital status (married or living as married vs. single (single, separated, divorced, widowed)), and education (< High school, High school, some college, ≥ college).

Table S2. Percent difference (95% confidence interval) in gestational urinary phthalates associated with food and beverage consumption habits, by race.[a]

	MEOHP			MEHHP		
	Overall (*n* = 395)	White (*n* = 197)	Black (*n* = 198)	Overall (*n* = 395)	White (*n* = 197)	Black (*n* = 198)
**Food & beverage consumption in general**						
Organic	−2.96 (−10.42, 5.13)	−6.76 (−17.30, 5.13)	1.01 (−9.52, 12.75)	−2.96 (−11.31, 6.18)	−6.76 (−18.13, 5.13)	0.00 (−11.31, 13.88)
Safe Plastics	2.02 (−4.88, 8.33)	−2.96 (−12.19, 7.25)	7.25 (−1.98, 16.18)	3.05 (−3.92, 11.63)	−0.99 (−11.31, 9.42)	9.42 (−1.98, 20.92)
Water: Soft Plastic	3.05 (−3.92, 11.63)	10.52 (−0.30, 23.37)	−4.88 (−14.79, 7.25)	3.05 (−4.88, 12.75)	10.52 (−1.00, 23.37)	−4.88 (−16.47, 9.42)
Water: Hard Plastic	1.01 (−6.76, 8.33)	−1.00 (−11.31, 11.63)	3.05 (−6.76, 12.75)	0.00 (−7.69, 9.42)	−0.99 (−12.19, 11.63)	3.05 (−8.61, 16.18)
Plastic Food Storage	2.02 (-4.88, 10.52)	3.05 (-7.69, 16.18)	0.00 (-9.52, 10.52)	2.02 (-5.82, 11.63)	2.02 (-9.52, 16.18)	1.01 (-9.52, 13.88)
Microwave Plastic	-1.00 (-6.76, 6.18)	0.00 (-9.52, 10.52)	-1.00 (-9.52, 8.33)	-1.00 (-7.69, 7.25)	0.00 (-9.52, 11.63)	0.00 (-10.42, 10.52)
**Food & beverage consumption during pregnancy**						
Organic	-1.98 (-11.31, 7.25)	-2.96 (-15.63, 10.52)	-1.00 (-12.19, 12.75)	-2.96 (-13.06, 7.25)	-4.88 (-17.30, 9.42)	-1.00 (-14.79, 15.03)
Unprocessed Food	1.01 (-7.69, 9.42)	-1.98 (-13.93, 11.63)	1.01 (-9.52, 13.88)	2.02 (-7.69, 12.75)	-1.00 (-13.93, 13.88)	3.05 (-10.42, 17.35)
Home Grown Food	8.33 (-2.96, 20.92)	3.05 (-13.06, 22.14)	9.42 (-4.88, 25.86)	8.33 (-2.96, 22.14)	4.08 (-13.06, 23.37)	9.42 (-6.76, 29.69)
Fresh Fruits & Veg	2.02 (-11.31, 18.53)	2.02 (-17.30, 25.86)	0.00 (-18.13, 22.14)	3.05 (-12.19, 20.92)	4.08 (-16.47, 31.00)	-1.00 (-22.12, 25.86)
Frozen Fruits & Veg	-4.88 (-13.93, 4.08)	-11.31 (-22.89, 1.01)	1.01 (-11.31, 16.18)	-5.82 (-14.79, 5.13)	-12.19 (-23.66, 1.01)	1.01 (-13.06, 18.53)
Canned Fruits & Veg	1.01 (−8.61, 10.52)	1.01 (−13.93, 17.35)	0.00 (−11.31, 11.63)	0.00 (−10.42, 11.63)	1.01 (−14.79, 18.53)	−1.98 (−13.93, 12.75)

NOTE: Grey shading indicates $p < 0.10$ for interaction by race. MEOHP, mono(2-ethyl-5-oxohexyl) phthalate; MEHHP, mono(2-ethyl-5-hydroxyhexyl) phthalate.
[a] Adjusted for age (years), BMI (kg/m^2), marital status (married or living as married vs. single (single, separated, divorced, widowed)), and education (< High school, High school, some college, ≥ college).

Table S2. Percent difference (95% confidence interval) in gestational urinary phthalates associated with food and beverage consumption habits, by race.[a]

	MEP			MMP		
	Overall (n = 395)	White (n = 197)	Black (n = 198)	Overall (n = 395)	White (n = 197)	Black (n = 198)
**Food & beverage consumption in general**						
Organic	−10.42 (−29.53, 13.88)	−16.47 (−39.95, 16.18)	−10.42 (−36.24, 25.86)	−7.69 (−18.13, 5.13)	−6.76 (−22.12, 12.75)	−11.31 (−23.66, 4.08)
Safe Plastics	11.63 (−10.42, 37.71)	24.61 (−7.69, 68.20)	−1.98 (−28.82, 33.64)	6.18 (−5.82, 18.53)	0.00 (−15.63, 18.53)	13.88 (−1.00, 32.31)
Water: Soft Plastic	5.13 (−14.79, 29.69)	4.08 (−19.75, 36.34)	1.01 (−30.93, 46.23)	9.42 (−1.98, 22.14)	7.25 (−7.69, 24.61)	4.08 (−12.19, 24.61)
Water: Hard Plastic	−7.69 (−24.42, 12.75)	−25.92 (−42.88, −4.88)	27.12 (−5.82, 71.60)	−1.00 (−10.42, 9.42)	−8.61 (−21.34, 6.18)	10.52 (−3.92, 27.12)
Plastic Food Storage	−4.88 (−22.89, 17.35)	−2.96 (−27.39, 29.69)	−5.82 (−30.93, 27.12)	3.05 (−7.69, 15.03)	6.18 (−10.42, 24.61)	−2.96 (−15.63, 11.63)
Microwave Plastic	11.63 (−9.52, 36.34)	16.18 (−11.31, 52.20)	5.13 (−22.89, 43.33)	4.08 (−6.76, 16.18)	2.02 (−12.19, 18.53)	1.01 (−12.19, 17.35)
**Food & beverage consumption during pregnancy**						
Organic	7.25 (−12.19, 31.00)	13.88 (−13.06, 49.18)	5.13 (−22.12, 41.91)	1.01 (−8.61, 10.52)	3.05 (−9.52, 18.53)	−1.98 (−14.79, 11.63)
Unprocessed Food	11.63 (−7.69, 34.99)	6.18 (−18.94, 37.71)	15.03 (−12.19, 50.68)	1.01 (−7.69, 10.52)	−0.99 (−13.93, 13.88)	2.02 (−9.52, 15.03)
Home Grown Food	8.33 (−14.79, 36.34)	−13.93 (−38.12, 20.92)	33.64 (−3.92, 84.04)	−1.00 (−12.19, 10.52)	−4.88 (−20.55, 12.75)	2.02 (−12.19, 17.35)
Fresh Fruits & Veg	−28.11 (−47.80, −1.00)	−25.17 (−50.84, 13.88)	−26.66 (−55.51, 19.72)	−13.06 (−25.17, 2.02)	−25.17 (−39.35, −6.76)	4.08 (−16.47, 29.69)
Frozen Fruits & Veg	−8.61 (−25.17, 11.63)	3.05 (−21.34, 34.99)	−22.89 (−43.45, 4.08)	−5.82 (−14.79, 4.08)	−12.19 (−23.66, 1.01)	0.00 (−13.06, 15.03)
Canned Fruits & Veg	4.08 (−15.63, 27.12)	11.63 (−18.94, 52.20)	3.05 (−21.34, 36.34)	4.08 (−5.82, 15.03)	5.13 (−10.42, 23.37)	2.02 (−9.52, 15.03)

NOTE: Grey shading indicates $p < 0.10$ for interaction by race. MEP, mono-ethyl phthalate; MMP, mono-methyl phthalate.

[a] Adjusted for age (years), BMI (kg/m^2), marital status (married or living as married vs. single (single, separated, divorced, widowed)), and education (< High school, High school, some college, ≥ college).

Table S2. Percent difference (95% confidence interval) in gestational urinary phthalates associated with food and beverage consumption habits, by race.[a]

	∑DEHP			∑DBP		
	Overall (n = 395)	White (n = 197)	Black (n = 198)	Overall (n = 395)	White (n = 197)	Black (n = 198)
**Food & beverage consumption in general**						
Organic	−3.92 (−17.30, 10.52)	−10.42 (−27.39, 10.52)	1.01 (−18.13, 23.37)	−4.88 (−16.47, 8.33)	−13.06 (−28.82, 7.25)	0.00 (−13.93, 16.18)
Safe Plastics	7.25 (−6.76, 22.14)	2.02 (−15.63, 23.37)	19.72 (−1.00, 43.33)	5.13 (−6.76, 18.53)	6.18 (−11.31, 28.40)	8.33 (−5.82, 24.61)
Water: Soft Plastic	10.52 (−2.96, 25.86)	12.75 (−4.88, 33.64)	8.33 (−13.06, 36.34)	9.42 (−2.96, 23.37)	7.25 (−9.52, 25.86)	2.02 (−13.93, 20.92)
Water: Hard Plastic	3.05 (−8.61, 16.18)	3.05 (−12.19, 20.92)	3.05 (−13.93, 23.37)	0.00 (−10.42, 11.63)	−6.76 (−20.55, 9.42)	12.75 (−1.98, 28.40)
Plastic Food Storage	−1.98 (−13.93, 11.63)	−1.00 (−17.30, 19.72)	−6.76 (−22.89, 11.63)	7.25 (−4.88, 20.92)	12.75 (−5.82, 34.99)	1.01 (−12.19, 16.18)
Microwave Plastic	−1.00 (−12.19, 12.75)	0.00 (−15.63, 18.53)	−2.96 (−18.94, 17.35)	−5.82 (−16.47, 5.13)	−6.76 (−21.34, 9.42)	−9.52 (−21.34, 4.08)
**Food & beverage consumption during pregnancy**						
Organic	−3.92 (−14.79, 9.42)	−7.69 (−22.12, 9.42)	2.02 (−15.63, 22.14)	−2.96 (−13.06, 8.33)	1.01 (−13.93, 17.35)	−3.92 (−17.30, 12.75)
Unprocessed Food	3.05 (−7.69, 16.18)	0.00 (−15.63, 18.53)	5.13 (−11.31, 24.61)	3.05 (−6.76, 15.03)	−6.76 (−20.55, 8.33)	17.35 (2.02, 33.64)
Home Grown Food	11.63 (−3.92, 28.40)	5.13 (−14.79, 29.69)	11.63 (−8.61, 37.71)	8.33 (−4.88, 23.37)	−6.76 (−22.89, 13.88)	16.18 (−1.98, 37.71)
Fresh Fruits & Veg	5.13 (−13.06, 28.40)	7.25 (−18.13, 40.49)	0.00 (−25.92, 33.64)	−9.52 (−23.66, 8.33)	−7.69 (−28.11, 17.35)	−9.52 (−29.53, 16.18)
Frozen Fruits & Veg	−5.82 (−17.30, 6.18)	−13.93 (−28.11, 2.02)	2.02 (−15.63, 23.37)	−5.82 (−16.47, 5.13)	−6.76 (−20.55, 9.42)	−5.82 (−19.75, 10.52)
Canned Fruits & Veg	0.00 (−12.19, 13.88)	2.02 (−16.47, 24.61)	−2.96 (−18.13, 15.03)	−0.99 (−12.19, 10.52)	10.52 (−7.69, 32.31)	−8.61 (−21.34, 5.13)

NOTE: Grey shading indicates $p < 0.10$ for interaction by race. ΣDEHP, sum of MEHP, MEOHP, and MEHHP in nmol/L; ΣDBP, sum of MBP and MiBP in nmol/L.
[a] Adjusted for age (years), BMI (kg/m^2), marital status (married or living as married vs. single (single, separated, divorced, widowed)), and education (< High school, High school, some college, ≥ college).

Table S2. Percent difference (95% confidence interval) in gestational urinary phthalates associated with food and beverage consumption habits, by race.[a]

	∑RPF		
	Overall (*n* = 395)	White (*n* = 197)	Black (*n* = 198)
**Food & beverage consumption in general**			
Organic	−5.82 (−16.47, 6.18)	−15.63 (−30.23, 2.02)	1.01 (−12.19, 15.03)
Safe Plastics	6.18 (−5.82, 18.53)	7.25 (−9.52, 27.12)	9.42 (−2.96, 24.61)
Water: Soft Plastic	9.42 (−1.98, 22.14)	7.25 (−7.69, 25.86)	5.13 (−9.52, 22.14)
Water: Hard Plastic	1.01 (−8.61, 11.63)	−6.76 (−18.94, 8.33)	12.75 (0.01, 27.12)
Plastic Food Storage	1.01 (−9.52, 12.75)	6.18 (−10.42, 24.61)	−5.82 (−17.30, 6.18)
Microwave Plastic	−0.99 (−11.31, 9.42)	−0.99 (−15.63, 15.03)	−4.88 (−16.47, 7.25)
**Food & beverage consumption during pregnancy**			
Organic	0.00 (−9.52, 11.63)	−0.99 (−14.79, 15.03)	5.13 (−9.52, 20.92)
Unprocessed Food	7.25 (−2.96, 18.53)	−1.98 (−15.63, 13.88)	17.35 (4.08, 33.64)
Home Grown Food	6.18 (−5.82, 20.92)	−2.96 (−19.75, 17.35)	9.42 (−5.82, 28.40)
Fresh Fruits & Veg	−7.69 (−22.12, 9.42)	−4.88 (−25.17, 19.72)	−12.19 (−30.23, 11.63)
Frozen Fruits & Veg	−10.42 (−19.75, −1.00)	−13.93 (−25.92, 0.20)	−7.69 (−20.55, 6.18)
Canned Fruits & Veg	1.01 (−9.52, 12,75)	−10.52 (−6.76, 32.31)	−6.76 (−18.13, 6.18)

NOTE: Grey shading indicates $p < 0.10$ for interaction by race. ΣRPF weighted sum of MBP, MiBP, MBzP, MEHP, MEHHP, MEOHP, and MEP in μg/L weighted by relative anti-androgen potency factors (RPF).

[a] Adjusted for age (years), BMI (kg/m^2), marital status (married or living as married vs. single (single, separated, divorced, widowed)), and education (< High school, High school, some college, ≥ college).

Table S3. Principal component analysis factor loadings.

		Food & beverage consumption in general						Food & beverage consumption during pregnancy					
		Organic	Safe Plastics	Water: Soft Plastic	Water: Hard Plastic	Plastic Food Storage	Microwave Plastic	Organic	Unprocessed food	Home grown food	Fresh Fruits & Veg	Frozen Fruits & Veg	Canned Fruits & Veg
**Overall**	PC1	0.80	0.73	−0.25	0.28	0.01	−0.17	0.82	0.55	0.40	0.62	0.09	−0.46
	PC2	−0.16	0.001	0.15	0.57	0.75	0.66	0.04	0.11	0.13	−0.06	0.47	0.08
	PC3	0.27	0.23	0.01	0.09	−0.40	−0.09	0.11	−0.23	0.41	−0.44	0.51	0.70
**White**	PC1	0.86	0.72	−0.36	0.15	−0.13	−0.47	0.82	0.53	0.31	0.67	−0.13	−0.62
	PC2	−0.15	−0.30	0.28	0.31	0.76	0.50	0.09	0.15	0.10	0.43	0.22	−0.48
	PC3	0.08	0.34	−0.39	0.61	0.17	0.09	0.11	−0.27	0.23	−0.32	0.66	0.32
**Black**	PC1	0.72	0.76	0.15	0.50	0.20	0.28	0.78	0.57	0.56	0.47	0.32	−0.08
	PC2	−0.45	0.03	0.19	0.45	0.82	0.65	−0.26	0.12	−0.22	0.00	−0.15	−0.08
	PC3	0.13	−0.18	0.25	0.18	0.04	0.16	−0.06	−0.11	0.32	−0.66	0.51	0.79

PC, principal component
